# Dynamic correlations between heart and brain rhythm during Autogenic meditation

**DOI:** 10.3389/fnhum.2013.00414

**Published:** 2013-07-31

**Authors:** Dae-Keun Kim, Kyung-Mi Lee, Jongwha Kim, Min-Cheol Whang, Seung Wan Kang

**Affiliations:** ^1^Institute of Complementary and Integrative Medicine, Medical Research Center, Seoul National UniversitySeoul, Korea; ^2^Division of Digital Media Engineering, Sang-Myung UniversitySeoul, Korea; ^3^College of Nursing, Seoul National UniversitySeoul, Korea

**Keywords:** EEG, HRV, heart coherence, meditation, lagged coherence, heart brain synchronicity

## Abstract

This study is aimed to determine significant physiological parameters of brain and heart under meditative state, both in each activities and their dynamic correlations. Electrophysiological changes in response to meditation were explored in 12 healthy volunteers who completed 8 weeks of a basic training course in autogenic meditation. Heart coherence, representing the degree of ordering in oscillation of heart rhythm intervals, increased significantly during meditation. Relative EEG alpha power and alpha lagged coherence also increased. A significant slowing of parietal peak alpha frequency was observed. Parietal peak alpha power increased with increasing heart coherence during meditation, but no such relationship was observed during baseline. Average alpha lagged coherence also increased with increasing heart coherence during meditation, but weak opposite relationship was observed at baseline. Relative alpha power increased with increasing heart coherence during both meditation and baseline periods. Heart coherence can be a cardiac marker for the meditative state and also may be a general marker for the meditative state since heart coherence is strongly correlated with EEG alpha activities. It is expected that increasing heart coherence and the accompanying EEG alpha activations, heart brain synchronicity, would help recover physiological synchrony following a period of homeostatic depletion.

## Introduction

The confluence of empirical neuroscience and computational network models is beginning to reveal the complex architecture of the human brain (Sporns, 2011; Thatcher, 2012). The various network of the brain during meditation processes transforming dysfunctional self-network to prosocial or transcendence network, have been proposed (Hasenkamp and Barsalou, 2012). The meditation has been categorized by focused attention and open monitoring (Lutz et al., 2008b). Attention network, network of brain regions typically involved in attention task, has proved to be activated through focused attention meditation (Mograbi, 2011; Froeliger et al., 2012). Self-networks, integrative fronto-parietal control network, and neurocognitive systems which consists of self-awareness, regulation, and transcendence during mindfulness meditation, covering both concentrative and open monitoring receptive process were also proposed (Vago and David, 2012). Another kind of meditation called loving kindness or compassion meditation has been shown to be related with emotion sharing network including limbic circuitry, insula, and anterior cingulated cortices (Lutz et al., 2008; Mograbi, 2011). A systematic review of mindfulness training showed that early phase of training are more concerned with the development of focused attention and the following phase are characterized by an open monitoring (Chiesa et al., 2011). The more detailed model specifying four sub-intervals in a meditative cognitive cycles was also proposed, which includes mind wandering (MW), awareness of MW, shifting of attention, and sustained attention (Hasenkamp and Barsalou, 2012). Activity in salience network regions were identified during awareness of MW and executive network regions were active during shifting and sustained attention. Brain regions associated with the default mode were active during MW.

The default network of the brain has been measured since the first recording of human surface EEG in 1932 (Berger, 1932) and early meditation studies has been conducted using EEG rather than fMRI or PET imaging (Cahn and Polich, 2006). As a network property, left prefrontal activity relative to right prefrontal activity, indicative of positive emotion, increased after meditation training period and that immune functions simultaneously increased in proportion with that activity (Davidson et al., 2003). Large amplitude gamma band activity with synchrony over various region of the brain has also been observed in expert Tibetan compassionate meditators (Lutz et al., 2004). An increase in alpha coherence in the whole brain region has been observed during relaxation meditation, whereas various non-ubiquitous topological patterns have been observed during meditation pursuing both relaxation and awareness simultaneously (Zelazo et al., 2007). Recently, cortical coherence patterns during various forms of meditation were uncovered in which the functional connectivity between brain regions coherence was significantly lowered during meditation compared to the rest (Lehmann et al., 2012).

As well as the pursuit of brain connectivity patterns or networks specific to meditation and linking the networks with the brain function, the peripheral index identifying the meditation has also been explored. Since Herbert Benson, a cardiologist at Harvard University, has shown the relationship between meditation and heart disease. He reported that meditation lowers blood pressure, physiological stress responses and, through accompanying system-wide physiological chain reactions, finally facilitates an overall bodily homeostatic effect, the so-called relaxation responses (Benson, 1975). Peripheral physiological changes during meditation, such as reduction in heart rate and/or systolic blood pressure, an increase in blood oxygen saturation, or a decrease in norepinephrine blood level, were still reported (Curiati et al., 2005; Travis et al., 2009; Gregoski et al., 2011). The various heart rate variability (HRV) indices during meditation were frequently reported (Phongsuphap et al., 2008; Libby et al., 2012).

A central theme of this paper is the relationship between the brain measures and the peripheral measure during meditation. Like earlier meditation studies in which specific brain location identifying state and trait of the meditation had been explored rather than the brain network (Cahn and Polich, 2006), the network analysis between central and peripheral measures during meditation has not been focused so far. Only a few attempts have been tried to associate brain with heart activities explaining mind-body connections (Takahashi et al., 2005). Associations between anterior cingulate cortex (ACC) theta activity and high frequency (HF) power in the HRV spectrum have also been reported (Tang et al., 2009). Recently, through functional neuro-imaging, it was reported that the coupling of heart rate and blood oxygen level-dependent (BOLD) signal in the ACC region was greater in meditative states than in neutral states (Lutz et al., 2009). These studies have suggested the presence of interactions between brain activities and peripheral activities, with such interactions especially observable during meditations.

As one of the novel peripheral index, heart coherence, a degree of regularity of the heart rate rhythm, has been found to be strongly related to slow breathing and positive emotion that are also core essence of the various meditation (Porges, 2007; McCraty et al., 2009; Thayer et al., 2009; Thompson et al., 2010). If we can find some connectivity pattern between the heart coherence and various brain indices, it would be helpful to find eventual mechanisms how the interactions between brain and heart facilitate the self-organization processes in the human body. In this study, brain indices determined by EEG alpha activities and heart activities determined by photoplethsmography (PPG) were measured and interactions between those two activities were assessed.

It is well known that alpha power represent the degree of the relaxation state and to what extent a subject is immersed in a meditative state, especially in the non-expert meditation (Chiesa, 2009). Alpha power also represent an cortical inhibition as an active process for information processing where ERD can be interpreted as release from inhibition and ERS (increase in alpha amplitude) reflecting the inhibitory aspect of alpha band oscillations (Klimesch, 2012). Meditation is an relaxation process as well as a kind of selective attention process to specific target while inhibiting irrelevant thought, so that source of EEG alpha activity could be considered as thalamo-cortical oscillations and cortico-cortical oscillations. Although, so far, there is no global brain theory in sight and the exact physiological mechanisms that generate alpha band activity are not yet known (Klimesch, 2012) there are some evidences of resting alpha power is positively associated with high performance brain function. Large resting alpha power may reflect a person's ability to build up a highly efficient attention filtering (Maclean et al., 2012), also predict healthy aging (Prichep et al., 2006; Roca-Stappung et al., 2012), can relieve various mental symptoms like pain, stress, anxiety (Huang and Charyton, 2008) whereas deficient alpha power were easily observed in developmental problem (Chan et al., 2007), pain(Huneke et al., 2012), etc^1^.

In that sense, we postulate EEG alpha activity as buffer capacity to enhance signal to noise ratio in attention filtering and to enhance relaxation from higher frequency oscillations, so that alpha activities has been intensively investigated throughout the paper. Furthermore, relative alpha power would be better index to integrate inter-subject difference than the absolute alpha power because it is normalized to 1. EEG coherence means to what extent do 2 channels of EEG share a common source, and also normalized to 1.

It has been used LF/HF as sympathovagal balance where LF (0.04–0.15 Hz) represent sympathetic activity and HF (0.15–0.4 Hz) represent parasympathetic activity of autonomic nervous system (ANS) (Task-Force, 1996)^2^. However, this conventional LF index did not consider activity of baroreflex and collective evidence heavily call into question the model of autonomic balance including LF HRV index as a biomarker of sympathetic activity (Eckberg, 1997; Heathers, 2012). In preliminary analysis for the same data sets in this paper, conventional HRV index did not significantly change during meditation compared to the baseline. For example, HF represents a parasympathetic activity which facilitates a deep relaxations but the HF also did not change during meditation. However, heart coherence was significantly changed in the preliminary analysis, so that heart coherence was selected for a measure representing heart activities during meditation. Therefore, the heart coherence and interactions between heart coherence and EEG alpha activities was also intensively investigated.

## Methods

### Participants

The central observation of this study is Autogenic meditation. A total of 13 autogenic meditators (*F* = 7, *M* = 6) were assessed (*M* = 43.5 ± 7.9 years, range = 29–59 years). These individuals had completed the 8 weeks autogenic standard training course which is the basic course of Autogenic meditation, in other words, Autogenic training (AT). AT is a self-help relaxation technique that was invented by a German psychiatrist named Johannes Schultz (Schultz, 1969) and further developed by Luthe (1969). The AT technique consists of six standard exercises. The first exercise aims at muscular relaxation, which is achieved mainly by repeating a verbal formula to encourage heaviness. Subsequently, the concentration is focused passively on feeling warm, then calming the cardiac activity, slowed respiration, warmth in the abdominal region and finally coolness in the head. The technique is usually learnt in groups over a period of 8 weeks and home practice of the exercises at least three times daily is encouraged (Kanji et al., 2006). All participants had been meditating daily at least for 1 month after they have finished the course (*M* = 0.8 ± 0.5 year, range = 0.1–2.0 years). Participants were recruited from a local autogenic meditation community through word of mouth and email. The experimental procedure was approved by the Research Ethics Committee of the Seoul National University Hospital and informed consent was obtained from each participant prior to commencement of the study.

### Recording conditions

EEG data were collected by Brainmaster Discovery 24E Digital EEG system with a 19-channel ECI electrode cap from the following locations: Fp1, Fp2, F3, Fz, F4, F7, F8, T3, C3, Cz, C4, T4, T5, P3, Pz, P4, T6, O1, and O2. These scalp locations were referenced to the linked ear lobes, with the ground at the AFz. Impedances were kept below 10 kΩ. The signals were recorded with a band pass of 0.43–80 Hz and a digitization rate of 256 Hz. To monitor real-time ANS activity simultaneously with EEG data, a photoplethysmographic (PPG) sensor was attached over the index finger of the left hand by means of a flexible Velcro strap.

### Procedure

The participants were instructed to sit on cushions and rest for 5 min for baseline measurements. The participant kept their eyes closed during the rest condition to compare that state with meditative state. And then meditate within the autogenic meditation process over a flexible period of time depending on their subjective meditation confirmation. (*M* = 8.2 ± 3.6 min, range = 3–15 min). We have designed the study for the meditators in order not to give any sense of restraint, but to give a natural environment. In that context, we did not predetermine the duration of meditation and let them finish the meditation according to their subjective experience whether they were in the state sufficiently as they had trained in the standard meditation course.

### EEG analysis in the alpha band

The EEG data from each of the recording were first visually inspected with transient muscle- and movement-related artifacts removed. An extended independent component analysis (ICA) algorithm was then run on the data using the runica algorithm within the EEGLAB software from Matlab (Delorme and Makeig, 2004). The resultant independent components that were related to horizontal and vertical eye movements were then marked and removed from the data.

EEG power spectra were then computed by using a fast-Fourier transform (FFT) based on 1024 points that correspond to 4 s epochs with a resolution of 0.25 Hz. Frequency bands were defined as follows: delta (δ), 1.0–4.0 Hz; theta(θ), 4.0–8.0 Hz; alpha(α), 8.0–12.0 Hz; and beta(β), 12.0–32.0 Hz. Relative alpha power was averaged for the 19 channels.

EEG coherence was computed for all 171 intrahemispheric and interhemispheric pair-wise combinations of electrodes. Coherence was defined as:

(1) $$\Gamma_{XY}^{2}(f)=\frac{{\text{G}}_{XY}{\left(f\right)}^{2}}{{\text{G}}_{XX}(f){\text{G}}_{YY}(f)}$$

where *G_xy_(f)* is the cross-power spectral density and *G_xx_(f)* and *G_yy_(f)* are autopower spectral densities for each channel *X* and *Y*, respectively. The *G_xy_(f)* is the sum of volume conduction and true connectivity, so that volume conduction corrected coherence also defined as:

(2) $$\text{LagCoh}(f)=\frac{{\left[{\text{ImG}}_{XY}(f)\right]}^{2}}{G_{XX}(f)G_{YY}(\text{f})-{\left[\text{Re}G_{XY}(f)\right]}^{2}}$$

The volume conduction corrected coherence was also referred to as zero phase lag removed coherence and lagged coherence (Nolte et al., 2004; Pascual-Marqui, 2007). The coherence used in this paper was the lagged coherence calculated from (2), not the coherence including volume conduction. The coherence was averaged for all pair-wise 171 combinations of the 19 channels for the alpha band.

One minute at the start of each measurement was excluded from the analysis since heart index cannot be calculated at the start of measurements. Subsequently, 4 s of EEG data were epoched successively without overlapping of epochs. Relative power, peak power, peak frequency, and coherence were calculated in alpha band for each epoch.

The EEG data for one participant was excluded due to technical problems. Thus, data analysis was performed on EEG results from 12 participants.

### HRV analysis

HRV time series was from PPG data by using a peak detection algorithm. Power spectral density was obtained from the FFTs. Heart coherence, a novel index (McCraty et al., 2009), was formulated as follows:

HeartCoherence=PeakPower/TotalPower,

where peak power was determined by calculating the integral in a window 0.03 Hz wide, centered on the highest peak in the 0.04–0.4 Hz range of the HRV power spectrum, and the total power was determined by calculating the integral in a window of 0.0033–0.4 Hz wide. Heart coherence has a value between 0 and 1 and indicates the magnitude of similarity between the waveform of the HRV tachogram and a sinusoidal wave.

Sixty seconds of HRV tachogram data were epoched successively from the start of measurements with an overlap of 56 s. Heart coherence were calculated for each 60 s epoch obtained every 4 s, within which alpha band EEG parameters were also calculated, in the manner described above for the same epoch. All calculations were performed by using Matlab 7.13.0 (Mathworks, USA).

### Individual statistical analysis

Heart coherence, relative alpha power, peak alpha power, alpha peak frequency, and average alpha coherence were calculated at each 4 s interval for each participant. Generally, steady-state EEG signal can be modeled by each 2–4 s consecutive epochs since longer epoch cannot satisfy stationarity, so that we could assume each consecutive 4 s EEG epochs used in this research as independent samples (Barrett et al., 2012; Nicolaou et al., 2012). The number of samples in the 5-min baseline ranges from 55 to 60 (mean = 59), and the number of samples in the meditation ranges from 25 to 220 (mean = 103) for the 12 participants. Mean recruiting more than 25 independent data samples definitely have Gaussian distribution irrespective of its original distribution, so that *t*-test can be applied for a comparison between baseline mean and meditation mean. Two-sample *t*-tests were performed to determine whether the mean values before meditation (baseline) and during meditation were significantly different for each of the participants. Significant differences were determined at a probability (*p*) level of 0.05. All data are represented as 95% confidence intervals with their corresponding *p*-values. Analyses were performed by using Matlab 7.13.0.

### Group statistical analysis

To perform group-based statistical analyses, HRV and EEG variable values for all individuals combined were determined at baseline and at meditation. Two-sample *t*-tests were performed to determine whether the variable values at baseline and during meditation were significantly different. Linear regression analysis were performed to determine if there were correlations between heart coherence and EEG alpha variables, both at baseline and during meditation. Regression coefficients for each condition and corresponding *p*-values are presented.

## Results

### Changes in heart coherence and EEG variables during meditation

**Figure 1:**
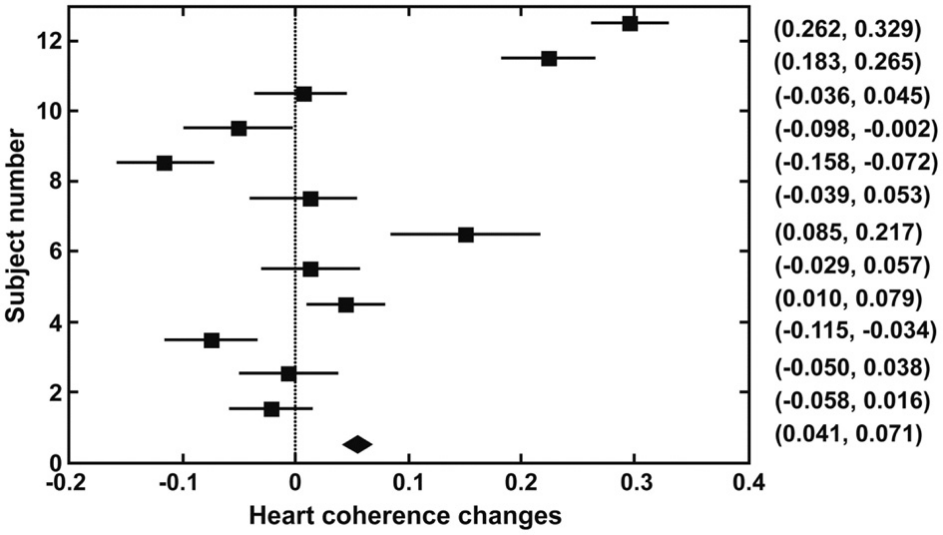
**Confidence intervals (95%) for changes in heart coherence from baseline to during meditation in each participant and for all participants combined (bottom indicates the result for all participants)**.

**Figure 2:**
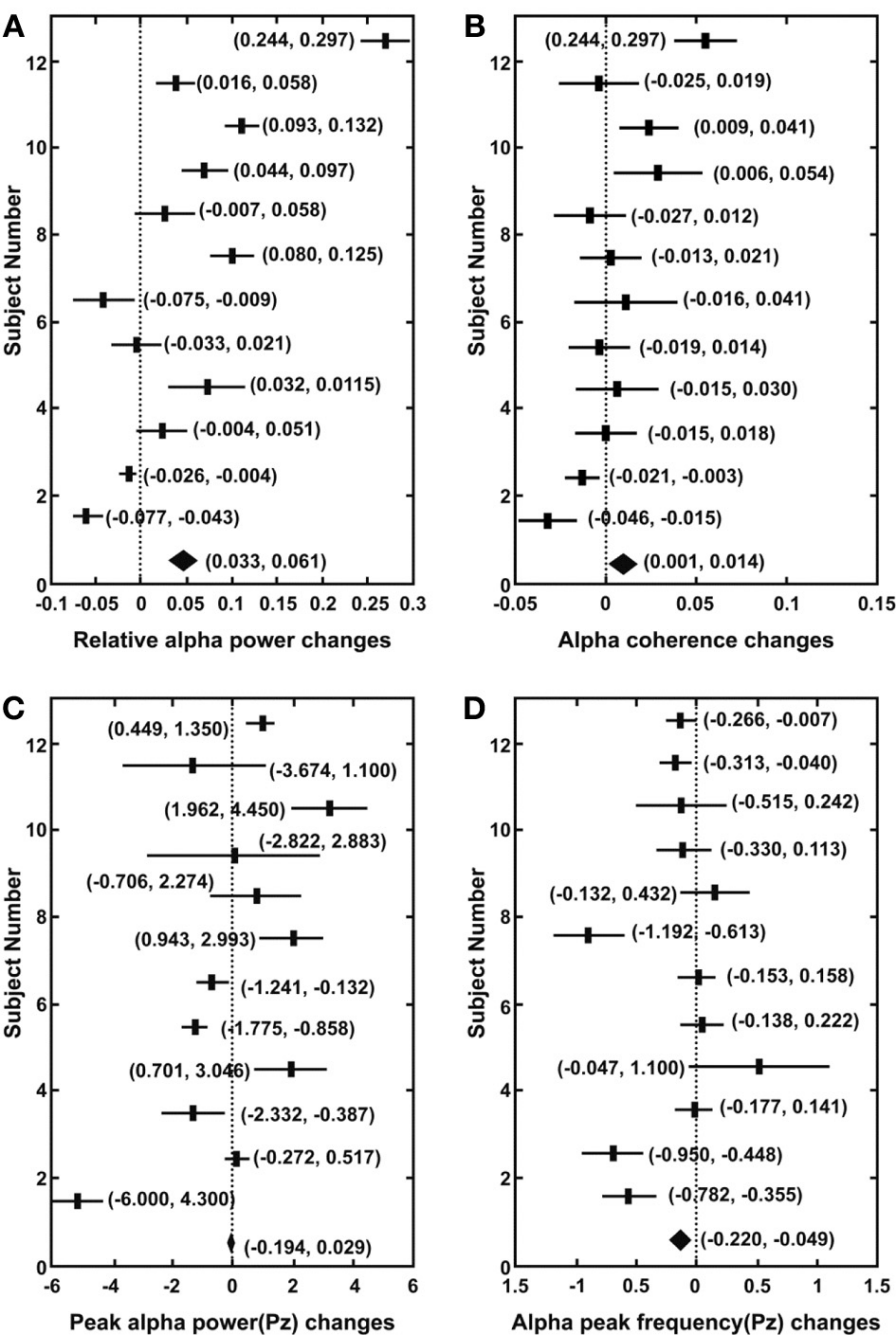
**Confidence intervals (95%) for changes in alpha band activities from baseline to during meditation in each participant and in all participants (bottom indicates the result for all participants). (A)** Relative alpha power averaged over 19 channels. **(B)** Alpha band lagged coherence averaged over 171 channel combinations. **(C)** Parietal peak alpha power (Pz). **(D)** Parietal peak alpha frequency (Pz).

Figure 1 shows the 95% confidence intervals of changes in heart coherence during meditation (duration 8.2 ± 3.6 min) compared to baseline (duration 5 min) period for each participant and for all participants. Figure 2 shows the 95% confidence intervals for changes in alpha band activities during meditation from that at baseline for each of the participant and for all participants. Table 1 provides a summary of the changes in heart coherence and EEG variables during meditation compared to levels at baseline for all participants combined. The table indicates the heart coherence significantly increased by 4.1 to 7.1% during meditations. Relative alpha power, which was averaged over 19 channels, also significantly increased by 3.3 to 6.6% during meditations. Similarly, alpha band coherence, averaged over 171 channel combinations, was also significantly increased 0.1 to 1.4% during meditation. Parietal alpha peak frequency, measured at electrode Pz, was significantly reduced by 0.049 to 0.22 Hz during mediation; however, there was no significant change in peak alpha power at electrode Pz during meditation.

**Table 1:** **Confidence intervals (95%) for changes in measured variables from baseline to during meditation for all participants**.

**Variables**		**Confidence interval of changes**
Heart coherence	↑	0.041 ~ 0.071 (*p* < 0.001)
Rel. alpha power	↑	0.033 ~ 0.061 (*p* < 0.001)
Alpha lagged coherence	↑	0.001 ~ 0.014 (*p* < 0.001)
Peak alpha power(Pz)	↑	−0.029 ~ 0.194 (*p* = 0.145)
Peak alpha frequency(Pz)	↓	−0.022 ~ −0.049 (*p* < 0.01)

### Correlations between heart coherence and EEG variables at baseline and during meditation

**Figure 3:**
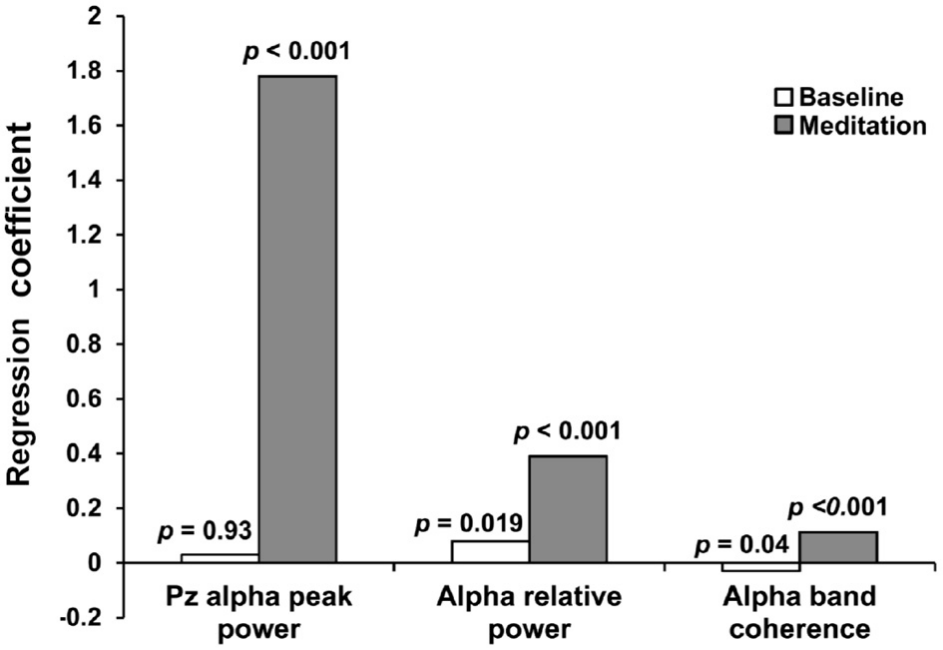
**Regression coefficients between heart coherence and EEG variables at baseline and during meditation for all participants**. Significant correlations between heart coherence and alpha variables was observed during meditation, but not in baseline. Alpha relative power was significantly correlated with heart coherence both during meditation and baseline. Regression coefficients for all EEG alpha variables were greater in meditation than in baseline.

Figure 3 shows regression coefficients between heart coherence and EEG alpha variables at baseline and during meditation for all participants combined. A significant correlation between heart coherence and alpha peak power was detected in during meditation (*R*^2^ = 0.066, *F* = 87.31 *p* < 0.0001; alpha peak power = 0.3 + 1.78 × Heart coherence), but not in baselines (*R*^2^ = 0, *F* = 0.0076, *p* = 0.93). The regression coefficient for the alpha peak power with respect to heart coherence during meditation was higher than baseline. Similarly a significant correlations between heart coherence and alpha band coherence was detected in during meditation (*R*^2^ = 0.074, *F* = 98.67, *p* < 0.001; alpha band coherence = 0.111 + 0.112 × heart coherence), but a significant negative correlations in baseline (*R*^2^ = 0.006, *F* = 4.04, *p* = 0.04; alpha band coherence = 0.157 − 0.032 × heart coherence). The regression coefficient for the alpha band coherence with respect to heart coherence during meditation was also higher than baseline. In contrast, there were significant correlations between heart coherence and relative alpha power during both periods, with the correlation level greater for during meditation (*R*^2^ = 0.149, *F* = 218.88, *p* < 0.0001; relative alpha power = 0.16 + 0.39 × heart coherence) than baseline (*R*^2^ = 0.0078, *F* = 5.52, *p* = 0.019; relative alpha power = 0.24 + 0.08 × heart coherence). The regression coefficient for the relative alpha power with respect to heart coherence during meditation was still higher than that of baseline.

## Discussions

### Heart coherence as a cardiac index of meditation and EEG alpha activities

Heart coherence, rather than conventional HRV indices, increased significantly during meditative states compared to baseline for all 12 participants. At the same time, whole brain EEG alpha activities, average relative alpha and alpha coherence also significantly increased while parietal peak alpha frequencies were significantly decreased. Heart coherence is a novel index which has not been explored so much in meditation research. Except volume conduction (zero-phase lag) removed lagged coherence adopted in this study, the enhancement of alpha power, alpha coherence and decrease of alpha peak frequency has been reported so long (Aftanas and Golocheikine, 2001; Kubota et al., 2001; Murata et al., 2004; Saggar et al., 2012). This could be inferred from the fact that meditative states, which generally concentrate on respiration or internal states, inhibit asynchronous cortical activities via prefrontal circuits; thus, synchronous alpha activations originating in the thalamus could affect the whole brain areas more actively (Thayer et al., 2009).

It has been reported that low frequency (LF) and/or HF power in the HRV power spectrum can increase during meditation (Phongsuphap et al., 2008; Wu and Lo, 2008, 2010). A HF power increase, indicative of parasympathetic activity, has been understood as a relaxation effect of meditation; however, a LF power increase has not been well explained. It seems confusing that LF power, representing sympathetic activity, can increase during meditation. Slow breathing in meditation induce cardio-respiratory synchronization, so-called the RSA (respiratory sinus arrhythmia), which makes LF power increase (Cysarz and Büssing, 2005; Ditto et al., 2006). This could be explained that such synchronization results in an increase in heart coherence suggesting that the peak power in the HRV power spectrum moves into the LF band and increase itself by slow and regular breathing during meditation. There are also another case showing respiratory influence on HRV index, in which the respiratory rate was fixed at 0.2 Hz and the resulting power changes did result in an increase in HF power (Takahashi et al., 2005). Despite the fact that cardio-respiratory resonant frequency varies from person to person, the frequencies are generally around 0.1 Hz. Thus, slow breathing is expected to increase the LF power in the HRV spectrum. On the other hand, an increase in HF power would be expected in deep relaxation meditation accompanying theta activations (Tang et al., 2009).

The LF index proposed by Task Force (Task-Force, 1996) has been recognized as indicator for the sympathetic activation and a LF/HF represents sympathovagal balance in many literatures, more than 300 publications indexed in Pubmed within the last year that explicitly refer to measures of sympathovagal balance derived from HRV metrics.(Montano et al., 1994; Susanna et al., 2013). But it is obvious that slow breathing enhance LF power (Eckberg, 1997; Heathers, 2012). In order to avoid confusion, careful consideration should be taken to separate sympathetic-activity-derived LF enhancement from cardio-respiratory-synchronized LF enhancement within slow breathing during meditation.

In this study, heart coherence, which reflects the degree of ordering in oscillations of heart rhythm intervals, is proposed as a cardiac marker for meditative states. The proposed heart coherence index would reflect both regular and slow breathing during meditation. In our study, the confidence interval for changes in heart coherence during meditations was 0.041–0.071 for all participants. Heart coherence seems to reflect the meditative states more clearly than the LF index.

It is generally known that positive emotion promote health. However, neuro-scientific substrates for the emotion mostly lie very deep in the brain so it is very difficult to quantify or modulate this area. However, it is well known that a breathing pattern for the negative emotions has a quite different from that for the positive emotions. The former one is shallower and more irregular while the latter is the deeper and more regular (Kim and Andre, 2008). The heart coherent states in which the heart rate accelerates during inhalation and decelerate during exhalation (Wundt, 1902; Porges, 2007) are more evident for the positive emotion, and the state was not observed with negative emotions. In other words, as emotions become more positive, the respirations become deeper at the same time. It is not clear and hard to determine whether positive emotions drive deep respirations or deep respirations drive positive emotions. However, heart coherence is closely related to the respiration pattern. The respiration pattern represents a healthy emotional state, and finally a healthy emotional state accumulates to promote health.

Heart coherence could be used as a meaningful index of a physiological marker of meditation that could easily be performed during our daily lives, if we consider our general experience that distracting thoughts and stressful conditions cannot coexist with slow and regular breathing. Furthermore, a heart coherence index extracted from heart rhythm intervals could be implemented in a simple and wearable device and used as a biofeedback modality for stress reduction. Therefore, heart coherence is a more appropriate index than the LF index for simple meditative states that are accompanied by slow and regular breathing.

### Relationships between heart coherence and EEG alpha band activities

In this study, relationships were assessed between the proposed heart coherence as a meditation index and EEG alpha band activities. The parietal peak alpha power is increased with increasing heart coherence during meditation but no such significant relationship was observed at baseline. Average lagged alpha coherence are increased with increasing heart coherence during meditation but reverse relationship was observed at baseline. Relative alpha power also increased with increasing heart coherence during both meditation and baseline while the regression coefficient still higher as similar in the other alpha variables during meditation than baseline.

There is a previous meditation-related study examining correlations between conventional HRV indices (LF, HF, LF/HF) and EEG variables (Takahashi et al., 2005); however, respiration rate in the study was fixed at predetermined rate that could give a sense of restraint to the participants. It would be more natural that the participant decides their respiration rate by themselves according to their own tempo for their successful meditative state.

There was a study exploring the correlation between average cardiac index changes and average EEG index changes during meditation (Kubota et al., 2001; Hamada et al., 2006) but there are few cases considering dynamic correlation changes between cardiac and EEG indices at within baseline and within meditation. One such exception was a study in which the correlation between heart rate and BOLD signals in the ACC region was higher in the meditative state than in a neutral state (Lutz et al., 2009).

In this study, the relationship between cardiac and EEG indices were explored at baseline and during meditation for 12 participants. The results show that our proposed cardiac index, heart coherence, has a significant positive correlation with every EEG alpha index (peak power, relative power, and average coherence) during meditation, but not during baseline.

At the same time, heart coherence had a stronger coupling, greater regression coefficient, with all EEG alpha variables during mediation than during baseline testing (Figure 3). The regression coefficient, slope of the regression equation between heart coherence and EEG alpha variables, means to what extent of EEG alpha activities changes when heart coherence increase by 1. If we only observed average changes in various variables, the dynamic correlations between heart coherence and alpha peak power, for instance, would not have been detected. Many participants did not show positive heart coherence changes when the average changes were statistically assessed (Figure 1). It means that coherent behavior of the heart rhythm was not so reliable for many of the participants. It means that many of the participants could not sustain heart coherent meditation for the entire duration of their meditation. However, it would be more natural that there were fluctuations in meditation quality within their entire meditation duration. Furthermore, all the participants only completed a basic course, and none were advanced meditator. Although heart coherence was not reliable in the most of the participants, there was strong coupling between heart coherence and EEG alpha variables within meditative state compared to the baseline. This could suggest that there are many moments in which heart coherence directly influence EEG alpha activities during meditation. In addition, alpha peak power did not also change significantly from baseline to during meditation in the analysis of group-based data. Regardless, the highest regression coefficient (Figure 3) during meditation compared to baseline, was between heart coherence and alpha peak power, indicates that there may be many moments when heart coherence is strongly coupled with alpha peak power during meditation, even though the relationship may not be detected during an entire meditation duration (Figure 2C).

An indication of meditation quality would be expected from the strength of the relationship, regression coefficient (Figure 3), between heart coherence and EEG variables. Heart coherence may not only be a cardiac index but also an index of meditation if heart coherence is strongly correlated with EEG alpha variables especially in meditation. However, there is also an evidence that heart coherence usually increases in the early phase of meditation, accompanying slow and deep breathing, and shows quite different patterns in the deep phase of meditation in which the heart coherence cannot represent such a meditative state any more (McCraty et al., 2009).

Although heart coherence cannot cover all stages or types of meditation, we anticipate that a heart coherence index will become a simple tool for quick assessment of Autogenic meditative states, which is easily achievable during people's daily lives, considering the fact that heart coherence can be implemented more easily than EEG index, based on the use of contemporary technologies.

In addition, all parameters observed in this study were indices reflecting a degree of ordering or self-organization; heart coherence, EEG alpha activities and the synchronizing relationship between heart coherence and EEG variables. Strengthening the degree of ordering by enhancing heart coherence, promoting EEG alpha activations and improving the relationship between heart and EEG variables would help in recovery of the homeostatic processes within our body. Interestingly, there were some researches showing harmonic frequency architecture in EEG study. Eleven Hertz alpha peak was clearly observed in vibrotactile discrimination task while harmonic peak at 22 Hz also emerged in a recent animal study (Haegens et al., 2011) and simultaneous appearance of 6 Hz frontal midline theta and 12 Hz alpha activity during retention period of a demanding working memory task in a human study (Jensen et al., 2002). Regarding the dynamic correlations between heart coherence and EEG alpha activities argued in this paper, we could assume that there could be a harmonic oscillation architecture in human body, connecting brain oscillating 10 Hz alpha frequency and heart oscillating 0.1 Hz respiration frequency (cardio-respiratory resonant frequency varies from person to person, the frequencies are generally around 0.1 Hz).

We still do not know how the heart coherence are coupled EEG alpha activity more actively in the meditation compared to the baseline. Further study will help to define this degree of ordering more clearly and a causality of the interactions between heart and brain more thoroughly and will eventually determine how we can achieve such a state more easily using physiological knowledge and biofeedback technologies.

### Conflict of interest statement

The authors declare that the research was conducted in the absence of any commercial or financial relationships that could be construed as a potential conflict of interest.

## Footnotes

^1^We have also observed many QEEG profiles having alpha deficiency in autism, mild traumatic brain injury, complex regional pain syndrome in author's clinic.

^2^LF HRV index is provoked in part by sympathetic nervous activity and also determined by activity of the baroreflex. For healthy population, LF induced by barorefelx activity could not be ignored.
